# User-Centered Design of a Mobile App for Weight and Health Management in Adolescents With Complex Health Needs: Qualitative Study

**DOI:** 10.2196/formative.8248

**Published:** 2018-04-04

**Authors:** Jordan Rivera, Amy C McPherson, Jill Hamilton, Catherine Birken, Michael Coons, Michelle Peters, Sindoora Iyer, Tessy George, Cynthia Nguyen, Jennifer Stinson

**Affiliations:** ^1^ Sunnybrook Health Sciences Centre Toronto, ON Canada; ^2^ The Hospital for Sick Children Toronto, ON Canada; ^3^ Bloorview Research Institute Holland Bloorview Kids Rehabilitation Hospital Toronto, ON Canada; ^4^ Department of Family and Community Medicine Faculty of Medicine University of Toronto Toronto, ON Canada

**Keywords:** obesity, weight loss, adolescent, mobile apps

## Abstract

**Background:**

Growing research has been conducted into the deployment and evaluation of mobile technology interventions for weight management in adolescents. However, no work has yet been conducted toward the development of these technologies for adolescents with complex health needs receiving specialized tertiary-level health care.

**Objective:**

The aim of this study was to conduct a user-centered needs assessment of adolescents interested in weight management with complex health needs requiring specialized health care services, their parents, and health care providers (HCPs) to inform the design and development of a mobile app for weight and health management.

**Methods:**

A qualitative study design was employed. Participants were recruited from two tertiary health care centers. Separate audiotaped focus group interviews were conducted with adolescents aged 12 to 18 years, parents, and HCPs. Interviews were transcribed, and field notes were collected by research staff. Iterative simple content analysis was performed independently by 4 research team members using computer software NVivo (QSR International) 10.0.

**Results:**

A total of 19 adolescents, 16 parents, and 21 HCPs were interviewed. Qualitative analysis revealed seven major themes related to app functionality: healthy eating, social support, self-monitoring, communicating with HCPs, supporting mental health, gamification and incentives, and user interface (UI) design. Adolescents provided several ideas related to each feature, whereas parents’ views focused on assistance with meal planning and greater access to HCPs. HCPs viewed the app as a novel and more acceptable platform to connect remotely with adolescents than conventional methods. They also strongly endorsed the value of social support capabilities and the ability to connect with an HCP.

**Conclusions:**

This is the first study to conduct a qualitative needs assessment in adolescents receiving specialized health care services toward the design of a mobile app for weight and health management. Our results indicate that core components of the app should include tailored meal recommendations and assistance with meal planning, social networking for peer support, customized and convenient tracking, remote access to HCPs, features to support mental health, and an attractive and engaging UI. These findings will be used to develop and evaluate a mobile app targeting adolescents with complex health needs.

## Introduction

### Background

Obesity in adolescents remains a major public health concern owing to its high prevalence and association with significant adverse health outcomes in adulthood [1]. Clinical interventions for obesity in children and adolescents aim to improve behaviors related to diet and physical activity (PA) and are based on psychological and family-centered theories of behavior change [2,3]. Adolescents with complex health care needs such as those with severe or complex obesity, disabilities, and other chronic health conditions may be excluded from such programs and generally show poorer rates of long-term success maintaining weight control [4-7]. Adolescents with complex health care needs often require specialized approaches to weight and lifestyle management that also address managing comorbid conditions, mental health problems, medications, and activity limitations [4,5,8-15]. Evidence-based behavioral strategies for weight management in children and adolescents with complex health care needs generally require more intensive counseling with an interprofessional health care team to help individuals and families build skills around goal setting, reducing sedentary time, and developing healthy eating habits through stimulus control and positive reinforcements [7,16]. Generally, factors that have been identified in successful programs are those taking place over 6 to 12 months, are family-centered in approach, and include group-based activities with peers [17]. Despite these advancements, the efficacy of emerging behavioral approaches is unclear, and behavioral interventions generally show modest reductions in body mass index (BMI) over the short-term [7].

### Information and Communication Technologies for Weight Management

Information and communication technologies have created opportunities to deliver accessible and cost-effective interventions for weight management that may help to sustain adolescents engaged in healthy lifestyle practices over the long-term. Mobile apps are being increasingly investigated for their potential to support long-term health management by providing more convenient and sustainable ways to implement health-related behavior change and access health care [18-20]. Mobile apps allow for users to track and interpret relevant health data, access information and resources, and to communicate with peer and health care provider (HCP) supports. Moreover, rapid advancements in remote monitoring capabilities and aggregated data analytics present new opportunities to develop more personalized, precise, and effective treatments for overweight and obesity [21]. Mobile apps can potentially serve as a medium to deliver accessible health care to young people in their community, as they comprise some of the most active users of this technology [22]. Moreover, adolescents of today are often described as digital natives having grown up surrounded by digital technology and demonstrate a natural capacity to process information in an electronic world [23].

A number of systematic reviews of technology-based interventions for overweight and obesity in adolescents found significant short-term (less than 6 months) reductions in BMI and improvements in dietary behaviors, PA, and self-monitoring [24-26]. Evidence is generally stronger for interventions employing mobile technology as an adjunct therapy to comprehensive weight management clinic-based programs [27-29]. Although these studies describe apps that have been used in research and health care settings, our group has conducted prior work to characterize the commercial app market for weight management apps and found that the majority of the apps available do not include evidence-based strategies and have not been developed with input from patients and HCPs [30]. These findings are supported by Shoffman and colleagues who reported on 57 commercial apps for pediatric obesity prevention and treatment and found that most lacked evidence-based recommendations [31].

Despite advancements made, a number of gaps persist in the literature. Minimal research has been conducted toward the development and evaluation of mobile health (mhealth) technologies for weight management, specifically targeting adolescents with complex health needs that require more frequent contact with HCPs and more intensive engagement with services [7,32]. Virtually no studies to date have been designed specifically for weight and health management in young people with severe or complex obesity, disabilities, and comorbid conditions, whose complex health needs often require specialized health care services and may not yet be properly represented in current research on health apps [33,34]. Furthermore, very little research has been conducted within health care settings and developed with patients and providers input toward the design and implementation of mhealth interventions. Finally, the majority of interventions have been developed for and tested in adult populations, and very few randomized controlled trials (RCTs) have been conducted in children and adolescents [25]. More research is needed to rigorously develop and evaluate mhealth interventions for weight and health management in young people with complex health needs requiring intensive and specialized treatment. The goal of this study was to conduct a user-centered needs assessment to inform the design of a mobile app for weight and health management targeting adolescents with severe or complex obesity, physical and developmental disabilities, and comorbid conditions receiving tertiary-level health care and in specialized clinical programs.

## Methods

### Study Design

A descriptive qualitative research design, as described in the study by Sandelowski [35], was conducted from 2015 to 2016. This research design was used for exploring the perspectives of adolescents with complex health care needs interested in weight management, as well as their parents and their HCPs regarding the essential features needed in a mobile app for weight and health management. Separate focus group interviews were conducted across two sites with adolescents, parents, and HCPs.

### Participant Recruitment

Purposive sampling of adolescents interested in weight management and healthy lifestyles was employed to collect information-rich cases that encompassed the diverse needs of a heterogeneous sample of adolescents with different health conditions and obesogenic risk factors. Participants in the age range of 12 to 18 years were recruited from two pediatric hospitals in Ontario, Canada. Participants at site 1 were recruited from a specialized tertiary care outpatient behavioral weight-management program for adolescents in the age range of 12 to 17 years with severe or complex obesity. Patients referred to this program have a BMI over the 99th percentile or a BMI over the 97th percentile and a significant obesity-related comorbidity requiring subspecialty care. Participants at site 2 were recruited from specialized inpatient units at a tertiary care pediatric rehabilitation hospital for adolescents in the age range of 12 to 18 years with disabilities, including acquired brain injury, neuromuscular, orthopedic and developmental disabilities. The study was approved by the Research Ethics Boards of both participating hospitals.

### Adolescent Selection

Eligible adolescents were introduced to the study by an HCP who was part of the client’s circle of care and could verify their capacity to consent. Adolescents were provided information letters describing the study and asked if they wanted their contact details passed onto the research team. Those interested were contacted by a research assistant who met with the adolescent to explain the study and obtain consent. Adolescents were deemed eligible to participate if they were receiving care at either recruitment site, were interested in weight and health management, and were able to speak and read English. Adolescents were excluded if they had severe cognitive impairments (eg, unable to take turns and consider other viewpoints), or a major comorbid psychiatric or medical illness that precluded their capacity to consent and ability to participate in a group discussion (eg, severe depression or anxiety), as identified by a member of their immediate clinical team.

### Parent and Caregiver Selection

Parents or caregivers of adolescents deemed eligible were also invited, though not required, to participate via the information letter. Parents or caregivers were required to speak and read English to participate. Parents were provided the option to participate with their spouse or on their own.

### Health Care Provider Selection

HCPs were introduced to the study by program managers and recruited from the respective clinical programs serving adolescent participants. No limitations were placed on health discipline or type of clinical service provided. Trainees were excluded from the study because of limited knowledge of the population.

### Interview Protocol

Study consent was obtained before each focus group interview. Adolescent and parent or caregiver participants completed two questionnaires on demographic and health characteristics, as well as level of use and comfort with mobile apps. HCPs completed one questionnaire on demographic and occupational characteristics. All interviews were conducted at the two hospital sites by four research team members, two of whom facilitated group discussion and two who observed and took field notes capturing verbal and nonverbal behaviors.

Focus groups were semistructured in format and followed an interview guide created by experts in pediatric obesity and informed by current research and clinical experience. Guides for adolescents and parents or caregivers included topics regarding their general experiences with weight management in their clinical programs, school, and home, before moving into more specific questions regarding desired app features and design. Adolescent and parent or caregiver focus groups were conducted concurrently and separately. The HCP interview guide similarly started with general questions about their openness to a mobile app related to healthy lifestyles before moving into more specific questions regarding app features and integration within their clinical practice. Sample interview questions are provided in Table 1.

### Data Analysis

Demographic data were coded and analyzed using Excel (Microsoft) to determine measures of central tendency and the distribution of values for the sample. All interviews were audiorecorded and transcribed verbatim, which were imported into NVivo 10.0 for data management. Qualitative content analysis was conducted using the approach described by Sandelowski, 2000 [35] using focus group transcripts and field notes. The data were analyzed to produce a simple descriptive summary of participants’ views, which is presented in everyday language [35]. More specifically, data for all participants were coded and organized into categories that reflected the emergent themes.

Three members of the research team coded the data (JR, TG, and MP). Study credibility and integrity were achieved using iterative questioning during interviews, frequent debriefing sessions with research team members, thick description, and prior examination of extant research in this area. Techniques were used to minimize the power differential between interviewer and respondents, including establishing rapport with ice-breaker questions using developmentally appropriate language, active listening, and relaxed body language. Data saturation was reached when no new codes were arising through iterative and open-ended questioning. The raw data were revisited regularly throughout the analytic process to ensure that the coding schemata reliably reflected the data. Discrepancies in coding were resolved with two senior members of the research team (JS and AM). Pseudonyms are used in place of participant names with quotes indicating the type of participant and the study site (site 1 or site 2).

**Table 1:** Sample interview questions.

Group	Broad questions	Program-specific questions
Adolescents	Can you tell me how long you have been working on healthy nutrition or physical activity goals and what it has been like for you? Why do you want to develop healthier lifestyle habits? Who do you feel should be involved with helping you to make and stick to healthy lifestyle changes?	What do you think about having a website and smartphone app that could help you to better manage your weight and develop a healthy lifestyle? Would you be interested in tracking your weight, what you eat, and your physical activities on an app? How about your mood, stress, sleep quality, and medications? Would you want a function on the app to help you set healthy lifestyle goals?
Caregivers	Can you tell me how long your child has been working on healthy nutrition or physical activity goals and what it has been like for him or her? Do you feel like your child has the tools or resources needed to manage and stick with their healthy lifestyle independently? Do you think your child wants to develop healthier lifestyle habits? Why or why not?	What do you think about having a website and smartphone app that could help your child to better manage their weight and develop a healthy lifestyle? Does your child keep track of their weight, what they eat, and their physical activities on a regular basis (eg, paper or electronic diary)? Why or why not? What information about managing your weight and developing a healthy lifestyle would you or your child want to learn more about to feel comfortable managing a healthy lifestyle?
Health care providers	What would you say are the patients’ and families’ biggest challenges and barriers to developing a healthier lifestyle? Do you feel like they have the tools or resources that they need to manage their healthy lifestyle independently?	What do you think about having a website and smartphone app that could help them to better manage their weight and develop a healthy lifestyle? How do you see an app fitting in with their care plan? Are there any other important features that we didn’t talk about that could help them to become healthier?

## Results

### Study Participants

A total of 56 participants were included in the study. Three focus groups were conducted with adolescents (n=19), four with parents or caregivers (n=16), and three with HCPs (n=21). A total of 34 participants were interviewed at site 1 (adolescents: n=12, parents: n=10, and HCP: n=12), and 22 were interviewed at site 2 (adolescents: n=7, parents: n=6, and HCP: n=9). Demographic characteristics of adolescents, parents or caregivers, and HCPs are presented in Tables 2-4. In general, most adolescents were female and in the age range of 12 to 16 years. The majority of parents were mothers in shared, middle income households. A range of HCPs were represented in our sample, including pediatricians, psychologists, social workers, registered nurses, dietitians, exercise coordinators, and occupational therapists.

### App Functionality

Thematic analysis revealed seven app functionalities of a mobile app for weight and health management, endorsed by adolescents, their parents, and HCPs. The seven desired app functionalities were as follows: healthy eating, social support, self-monitoring, communicating with HCPs, supporting mental health, gamification and incentives, and user interface (UI) design. Similarities and differences in perspectives were compared between participant groups and outlined below. All participant groups felt that a weight and health management app that is geared toward the unique needs of adolescents receiving specialized health care services to promote healthy weight-related behaviors would be beneficial.

### Key Functionality Components

#### Support for Healthy Eating

Adolescents identified a number of features that they felt were crucial to support healthy eating, including meal recommendations, recipes, nutrition information, and meal tracking. Adolescents desired meal suggestions to be personalized to factors such as mealtime patterns, social activities, and health conditions.

For example, one respondent proposed meal suggestions specific to popular restaurants or frequented eating locations, as illustrated in the following quote:

So like a list of the most popular restaurants...You could even have a section where it gives you options if you go to that place.

Lily, adolescent, site 1

Adolescents also desired a variety of new healthy recipes, as exemplified by the following quote:

Maybe food recipes is a big one, because we can eat healthy all we want but if we continue to eat the same grilled chicken breast, it will get boring. So maybe it could update all the time with different food recipes.

Sarah, adolescent, site 1

**Table 2:** Demographic characteristics of health care provider participants.

Characteristics		Health care providers (n=21)
**Type of health care provider, n (%)**		
	Pediatrician	2 (10)
	Psychologist	3 (14)
	Social worker	3 (14)
	Registered nurse	3 (14)
	Dietitian	2 (10)
	Exercise counselor	2 (10)
	Occupational therapist	6 (28)
**Sex, n (%)**		
	Female	21 (100)
Experience as a health professional (years), mean (SD)		11.2 (8.0)
Experience working with children (years), mean (SD)		8.21 (6.6)
Experience in obesity or weight management (years), mean (SD)		5.23 (6.6)

**Table 3:** Demographic characteristics of teen participants.

Characteristics		Teens (n=19)
**Gender, n (%)**		
	Female	13 (68)
	Male	6 (32)
Average age (years), mean (SD)		14.7 (2)
**Current grade, n (%)**		
	Grade 6	1 (5)
	Grade 7	3 (16)
	Grade 8	1 (5)
	Grade 9	2 (11)
	Grade 10	3 (16)
	Grade 11	4 (21)
	Grade 12	4 (21)
	College	1 (5)
**Work status, n (%)**		
	Not working	15 (79)
	Part-time work	4 (21)
**Medical conditions, n (%)**		
	None	7 (37)
	Not sure	7 (37)
	Asthma	3 (16)
	Autism	1 (5)
	Sickle cell	1 (5)

**Table 4:** Demographic characteristics of parent participants.

Characteristics		Parents (n=16), n (%)
**Type of caregiver**		
	Biological mother	14 (88)
	Biological father	1 (6)
	Adopted father	1 (6)
**Employment status**		
	Unemployed	2 (13)
	Part-time	2 (13)
	Full-time	11 (69)
	Retired	1 (6)
**Household income (CAN $)**		
	Less than 25,000	3 (19)
	25,000-49,000	1 (6)
	50,000-74,000	2 (13)
	75,000-99,999	1 (6)
	100,000-150,000	6 (38)
	Above 150,000	2 (13)
	Do not want to answer	1 (6)
**Education**		
	Some high school	2 (13)
	Graduated secondary school	1 (6)
	College or technical school	5 (31)
	Some graduate school	2 (13)
	Graduate degree	6 (38)
**Marital status**		
	Single	2 (13)
	Married or living common law	12 (75)
	Separated	1 (6)
	Divorced	1 (6)
**Ethnic background**		
	Aboriginal	0 (0)
	White	9 (56)
	South Asian	0 (0)
	Chinese	0 (0)
	Black	1 (6)
	Filipino	0 (0)
	Latin American	0 (0)
	Arab or West Asian	0 (0)
	Southeast Asian	0 (0)
	Korean	0 (0)
	Japanese	0 (0)
	Other (ie, Portuguese, Greek, or Italian)	3 (19)
	Do not want to answer	3 (19)

Related to this, nutrition information for specific foods was desired to facilitate more convenient grocery shopping. One adolescent stated the following:

You brought up using barcodes, maybe you could be able to go to the grocery store, scanning a barcode and the nutrition facts popping up and maybe give you more information than is on the package and see if that is in your list of things that you could have.

Megan, adolescent, site 1

The feedback provided by the app would ideally reflect their individualized healthy eating plan established between the adolescent and their health care team.

Adolescents and parents felt a meal-tracking feature would be beneficial, as a strategy for health-related behavior change, exemplified by the following quote from one adolescent:

Say you go out and eat something for fast food and we put it in [the app] and it notices that we have had that [food] too many times that it gives us a tip saying be careful.

Heather, adolescent, site 1

One parent provided a real-world example of how such a feature might provide guidance to their adolescent, as illustrated in the following quote:

A reminder...I’ve had hamburger and French fries, okay well then have a hamburger and French fries in 4-5 days but not tomorrow.

Susan, parent, site 2

Parents’ priorities centered primarily on assistance with meal planning. Parents desired an app that would provide nutrition information and suggestions for healthy meals personalized to their child. One parent stated the following:

Yeah, I think [meal planning] is the biggest thing. Or on the app what are the good snacks and the ingredients and what they need to do, just so they have everyday planned out.

Margaret, parent, site 1

Another parent wanted greater variety with food selection, as illustrated in the following quote:

What things you’ve discovered that you can eat other than apple sauce.

Lilian, parent, site 2

HCP’s responses centered on meal tracking and emphasized identifying adolescents’ personal health goals and adapting self-monitoring practices appropriately to promote healthy eating. One HCP stated the following:

At least get a push notification saying we noticed your weight has gone up, try eating more fruit, based on if they were entering data regarding their diet, or depending on what their goal was...to help people make that connection between knowing and actually implementing what they know.

Kathryn, HCP, site 2

#### Social Support

A key feature identified by the majority of adolescents was social support capabilities within the app. Social connection, or a way to maintain contact with peers, was frequently discussed as a critical feature for app use. One adolescent stated the following:

I think what you could actually have is something that could keep you connected to everyone else in the program...we have known each other for 10 weeks and it is kind of sad that we are not going to see each other anymore.

Lily, adolescent, site 1

An app could be used to prolong connection with peer social supports.

Encouragement and coping support were also discussed as potential benefits to a social support feature, which would allow for adolescents to share encouraging messages with peers and to seek out or offer advice regarding healthy lifestyles. For example, one adolescent commented the following:

Every once and a while you click on someone’s name and send them a little private message or a little motivation thing.

Sarah, adolescent, site 1

To facilitate social support, adolescents suggested linking to other social media platforms that are already in popular use, while also providing ways to engage in more private conversations with peers in similar situations (eg, participating in a weight management program). One adolescent stated the following:

It would be cool if we could have a section we could log into and it would just be for your group and there you could have a chat to stay connected.

Rebecca, adolescent, site 1

The importance of social support to adolescents’ motivation to adhere to healthy lifestyle change was reinforced by parents and HCPs who also identified social support frequently as a critical app feature. One HCP stated the following:

One thing we have heard back that people actually really like is the social [support].

Dana, HCP, site 1

Likewise, one parent commented the following:

Being here they’re with their own, at least I feel my daughter feels more herself. Being able to talk to people with the same issue.

Margaret, parent, site 1

HCPs and parents also endorsed using existing social media apps such as Instagram for motivation, entertainment, and encouragement, as illustrated in the following quote:

I’m really obsessed with the idea of having an Instagram with it...like look guys this is what [everyone] did today!

Maria, HCP, site 2

#### Self-Monitoring

Adolescents supported tracking data such as meals, PA, sleep, mood, and general health and that tracking be made customizable and related to personal goals. For example, one adolescent stated the following:

You’re able to pick from a list of things, like say that there is a list of 20 different things that you can track, but you pick maybe 8 of them.

Alice, adolescent, site 1

Adolescents also described how tracking would facilitate greater awareness of unhealthy lifestyle behaviors. One adolescent commented the following:

Maybe track how much sleep I need...When I’m feeling really tired I can’t [always] recognize it, it could tell me you need to go take a nap right now. Like when your body is really drained, go sit down.

Adolescent, site 2

HCPs similarly supported tracking as an appropriate technique for understanding lifestyle patterns and identifying areas for improvement. One HCP suggested the following:

Recording [and] tracking patterns, because lots of times our kids will say right after school [is when] they breakdown.

Jessica, HCP, site 1

However, both parents and adolescents expressed concerns that tracking aspects of health over the long-term can be difficult to sustain. One youth stated the following:

Just some personal experience with apps like this. I find that you start off really well with tracking, but overtime you forget or you don’t put in accurate information. So it is really easy when it comes to tracking to lose focus.

Lily, adolescent, site 1

Parents were particularly concerned that tracking all aspects of their life could be an emotional burden on adolescents, as was exemplified in the following quote:

[My daughter] tracked for a couple of years, and then she just had a meltdown. She refuses to track anything now. And my family doctor says she has every right not to want to track. Because they’re telling you not to eat and then to write down everything you did eat, like continuously in your face.

Margaret, parent, site 1

Finally, HCPs discussed the importance of setting realistic goals and considering the risk for unhealthy tracking, as demonstrated by the following quote by an HCP:

I think the danger is if they start using the app to expedite something. Their weight is going to be very tricky because we also want you to be comfortable with your weight. [Kathleen, HCP, site

Kathleen, HCP, site 1

#### Communicating With Health Care Providers

Adolescents liked the idea of being able to keep in touch and communicate with their HCPs within the app. When considering the different features that could potentially be offered by the app, adolescents identified multiple ways that they would consider using to communicate with HCPs, such as video calling, SMS text messaging (short message service, SMS), and sharing health data. One adolescent stated the following:

Virtual check-ins would be pretty cool, almost if we could make our own little appointments...they could have a time slot and then we could be like “oh I’m free” so then we just add our own appointment. And then we just FaceTime.

Dianne, adolescent, site 1

Adolescents endorsed the utility and convenience offered by remote access to interprofessional health care supports. This is demonstrated by one adolescent who suggested the following:

[Having] professionals you could talk to in case you have a problem or you’re feeling down...At that moment in time, they could advise you and give you some strategies to help you.

Nicky, adolescent, site 2

Furthermore, adolescents endorsed greater comfort and more efficient care with greater access to familiar HCPs, with one adolescent stating the following:

I think it would be cool to be able to connect with [our HCP] and ask them for advice because we have known the people we have been working with for a while. So it’s more comfortable asking them.

Lily, adolescent, site 1

Finally, data sharing with HCPs was suggested as many youths felt that this would provide a more accurate and up to date picture of their health, without constantly explaining what they were doing to maintain a healthy lifestyle. This was illustrated by a quote from an adolescent who stated the following:

Each time you go for your therapy, you wouldn’t have to explain how you’re feeling, or how you have been doing since the last time you saw them. You could just show them, or they could already have it written down.

Ian, adolescent, site 2

However, adolescents still desired control over privacy in their communication, with one adolescent stating the following:

There could be an option to make it anonymous, or a direct message, or if you want you could put it to the group chat.

Heather, adolescent, site 1

Parents also endorsed the benefits of having greater access to HCPs, whom many believed could provide needed emotional support to adolescents. One parent stated the following:

If they could talk with [a HCP], I think that would be the best thing, because sometimes [my daughter] just doesn’t want to talk to me, she gets all frazzled and then I start getting mad, and it’s just going to make it worse.

Mark, parent, site 1

HCPs were also open to communication with adolescents using an app and acknowledged adolescents’ comfort and adeptness with mobile apps, particularly as a communication tool over more traditional platforms such as email. One HCP stated that using an app can be “a way to communicate with [youth] that they are tapped into [and] that’s groundbreaking. They are like, why do you want my email, what are you, 100 [years old]?” (Nadia, HCP, site 1). HCPs also described how sharing health data over the app would facilitate more personalized, targeted and consistent care, as illustrated in the following quote:

...with the app it’s a nice kind of consistency. If I don’t [see] you all the time but you wrote on the app “I’m sad today” then I can get you that support. So maybe I can get you someone from psychology or somebody from recreation that you have a good bond with to come talk to you.

Jacklyn, HCP, site 2

However, HCPs still raised doubts about the practicality and enthusiasm held by adolescents for sharing their health data with their health care team, stating it would be “weird if you’re filling it out knowing a HCP is going to see it” (Tara, HCP, site 2).

#### Supporting Mental Health

Adolescents, parents, and HCPs all identified support for mental health as an important component of app function. Features that were suggested by adolescents included information about emotions, mood, and their impact on motivation, as well as healthy coping strategies such as relaxation, stress reduction, and building self-efficacy. Adolescents and HCPs both suggested inspirational messages, positive affirmations, reframing negative thoughts, and connecting with social supports as potential coping strategies facilitated by the app.

HCPs also viewed an app as an acceptable and useful tool for adolescents to self-monitor mental health, including mood and thought patterns, as exemplified by one HCP who stated the following:

You click on the app, first thing is “how are you feeling?”

Melissa, HCP, site 2

HCPs also felt that the app would allow adolescents to more honestly report their mood and its impact on their lifestyle. One HCP stated the following:

I think they’d be more willing to say it on the app as opposed to telling us “I’m not having a good day today.” I think they’d be more open to doing it that way than verbally.

Lindsay, HCP, site 2

Finally, parents and HCPs found it important that support also be provided to parents to promote mental wellbeing and positive self-image in their adolescent. One parent (Eleanor, parent, site 2). in particular claimed that her daughter’s mental well-being and self-image were the “most important things” to her, even over her daughter’s weight or adherence to diet. Another HCP (Darlene, HCP, site 2) suggested the “model of giving parents the tools they need to be the therapist with their adolescent. *”*

#### Gamification and Incentives

Adolescents proposed games and rewards-based incentives to help motivate continued use of the app. Suggestions included a point systems, monetary rewards, as well as multi-player gaming. HCPs also endorsed games and rewards, particularly group-based games as strong motivators for adolescents. One HCP stated the following:

I think that’s really motivating, when you have a goal in mind and you want to [improve] every single day. I think for kids that’s a good way to do it too. They’re always trying to compete against their classmates so I think having a goal oriented program would be good.

Patricia, HCP, site 2

#### User Interface Design

The most salient need in the UI design raised by adolescents was that it be attractive and entertaining to motivate its regular use. One adolescent’s quote encapsulates this notion, as stated in the following:

I feel like if we were to make an app, you have to make it entertaining...because even on the iPhone and the Android you automatically get a health app but almost no one ever touches it.

Julia, adolescent, site 1

Some adolescents compared the need to design the app to be interesting and engaging in a similar fashion to popular social media apps such as Snapchat and Instagram, which are accessed frequently throughout the day by many teens. HCPs also stressed that the app be simple and easy to use, preferably with minimal reading. One HCP stated the following:

Simplicity, just keeping it simple, so no food entry or that kind of stuff. That is what drives people away from using it.

Carmen, HCP, site 1

## Discussion

### Principal Findings

This qualitative study describes the perspectives of adolescents with severe or complex obesity or physical and developmental disabilities regarding the development of a mobile app to support weight and health management. Participants desired an app with features to enhance existing weight management practices and improve long-term maintenance of healthy behaviors. These features centered on healthy eating, social support, self-monitoring, communicating with HCPs, supporting mental health, gamification and incentives, and the UI design.

Healthy eating emerged across participant groups and included proposed features for meal planning, cooking, nutrition information, and more effective ways to self-monitor. The US Preventive Services Task Force considers a dietary component a critical aspect of comprehensive behavioral pediatric weight management programming [36]. However, a review of commercially available apps for weight management found only one-third included features to support healthy eating [30], whereas another review of apps specifically for the prevention of obesity in children found less than a third (27.4%) provided nutrition-related education, and only 11.2% assisted with meal planning [37].

Seventy-five percent of the top rated smartphone apps for weight loss are narrowly based on the energy-balance model of weight management and rely on a food database to track and control caloric intake [2,38,39]. In contrast, assistance with meal planning has not been widely reported in apps developed for weight management, particularly in young people [37]. Curtis et al have conducted preliminary work describing the design and theoretical development of a user-centered healthy eating app targeting parents for childhood weight management [40]. They identify time-saving and convenience with meal preparation as key characteristics desired by parents. Meal planning features such as recipes and nutrition information were strongly endorsed by both adolescents and parents in this study. Few apps targeting adolescents have been found to provide tailored assistance with meal planning and recipes for healthy eating, with most apps narrowly focused on dietary tracking and assessment [25,41]. Adolescents’ needs for healthy eating expanded beyond self-monitoring capabilities and included more convenient and efficient ways to plan meals and make healthier food choices throughout the day.

Self-monitoring, which involves tracking relevant health data and analyzing trends, is argued to be a central strategy of effective weight management [42,43] and is one of the most common strategies incorporated into technology-based interventions for weight, diet, and health management [25,44]. Electronic devices can provide more convenient and accurate ways to monitor personal health data than paper methods, thus increasing the sustainability of behavior change [45]. The potential effectiveness of emerging digital technologies for self-monitoring and health management is based on the proposed capabilities for more comprehensive and precise measurement, allowing for more personalized feedback to be generated [2].

Studies of mobile device interventions in adults have demonstrated significant associations between frequency of dietary and PA self-monitoring and weight and health-related outcomes [44,46]. Cartel et al reported that those with the highest frequency of mobile app use for self-monitoring had a −6.4 kg lower follow-up weight at 6 months than those with the lowest frequency of app use. Studies in adolescents have shown mostly mixed findings [25], likely attributable to a lack of longer-term trials demonstrating the effectiveness of mobile device interventions beyond 12 months, as well as to large variability in intervention designs and adherence rates to mobile device health tracking [43]. Participants in our study perceived difficulties in sustaining tracking over the long term, but believed the information would benefit their understanding of their health and support making healthier lifestyle choices. Self-monitoring adherence remains a major problem with current mobile devices for weight loss [43]. Our findings suggest an app would be more effective and engaging if tracking was made personally relevant and tied to personal goals, thereby facilitating greater awareness and evaluation of goal attainment and reinforcing behavior change.

Social support was seen as an important app feature for adolescents to stay connected with peers they had met through their hospital. Peers were valued as major sources of motivation, confidence, and learning. Our findings reinforce the value of social support and relationships to adolescents’ adoption of health beliefs and behaviors [47,48], including dietary and PA behaviors [49-53]. Smartphones and social media apps have reshaped how adolescents communicate with different peer groups on health-related topics [47]. The application of social media in health interventions has been examined in a systematic review of online social networks, which found mostly small and nonsignificant results [54]. These studies, conducted mainly in adults, used commercial websites such as Facebook and Twitter, as well as researcher-developed, health-focused social networks. Researcher-developed social networks were shown to be more effective for the users they retained over a period of time, suggesting that online social networks for health behavior change would be more useful for individuals already contemplating behavior change and are highly motivated [54].

Fostering supportive social relationships is a key strategy in pediatric clinic-based weight management programs, particularly in the maintenance phase of treatment [53]. The implications for a social support functionality in a health app for adolescents with complex health needs include the potential for online social networks to enhance motivation and adherence with healthy lifestyle changes. In addition, social network apps can allow greater access to social supports that are known to positively influence health behavior change. For many adolescents in the study, supportive relationships were central drivers of change, thus creating opportunities to leverage existing social networking applications and technologies to enhance and sustain health behavior change.

Communication with HCPs using a mobile device has the potential to significantly expand access to professional support and reflects ongoing initiatives within telehealth to develop applications for remote health care delivery and monitoring. Although efforts to harness mobile technologies for remote health care delivery are growing rapidly [55], our findings expand on the perceived utility of these technologies not only for enhancing access to health care but also to enhance continuity of care between patients and providers most knowledgeable about their health. Several adolescents viewed their HCPs as important resources for information about nutrition and healthy meal ideas, as well as for support during times of emergency or crisis. They ascribed greater comfort and openness to interacting remotely with providers they are familiar with regarding their progress and setbacks with weight management, suggesting that maintenance of the therapeutic relationship can serve as a major driver of adolescents’ adherence to healthier lifestyle change.

Telehealth interventions to extend therapeutic contact for children and adolescents with overweight and obesity have incorporated telephone, email or Internet, and mobile device SMS [56-61] The underlying aim and rationale of these technologies is to improve health care service coordination and continuity through client education, health information transfer, and real-time assistance with self-management [62]. More recently, [25] systematically reviewed studies of mobile device technologies for adolescent weight management and describe pilot RCTs by Nguyen et al [63] and Patrick et al [64]. Nguyen and colleagues [63] employed SMS, email communication, and telephone coaching for adolescents with overweight or moderate obesity to connect them with a registered dietician who was trained to provide additional therapeutic support. In the study by Patrick et al the researchers also used SMS, Internet, and telephone calls to provide adolescents at risk for type 2 diabetes access to a health counselor for support. Neither study found significant effects of the intervention on weight loss. However, Nguyen and colleagues point out there are few theories or frameworks to inform the structure, frequency, and duration of communication with HCPs [63]. Turner et al [25] in their review describe several usability and design studies showing improvements in diet, sedentary behavior, adherence to treatment, and self-monitoring with remote access to HCPs. Large-scale RCTs of differing systems and arrangements outlining remote HCP communication for adolescents are needed to better inform design of an optimal program structure.

The implication of our findings on future research and development of mobile device health provider communication suggests such a feature would be more effective when used by patients and their regular health providers with whom trust and a therapeutic alliance has been established. This way, more personalized and meaningful support can be provided, particularly for adolescents with complex health care needs. Furthermore, protecting privacy and ensuring security of personal health information is paramount to the utility, effectiveness, and safety of any mobile health intervention. Ultimately, our findings suggest that a mobile app can enhance or expand an existing health care service arrangement, rather than establish a new arrangement, and must be appropriately, safely, and securely implemented. Adolescents and parents view their HCPs as important sources of information and support and would benefit from easier access to them.

Support for mental health incorporated ways to feel motivated and increase self-efficacy. Adolescents, parents, and HCPs discussed the importance of emotion, motivation, and positive self-image for weight management success. Adolescents specifically touched on managing stress and mood as important components to making healthier lifestyle changes. Some specific strategies related to supporting mental health using the app included guided relaxation and mindfulness practices, access to personal supports, and speaking to an HCP. HCPs suggested storing affirmation statements, distraction techniques, and behavioral activation (eg, prompts) be incorporated into the app. Many of these strategies reflect components of emerging medical and commercial apps that have attempted to employ psychological theories into their design, mostly centered on cognitive behavioral therapy techniques such as mindfulness, relaxation, and emotional control, as well as providing tailored recommendations and information related to mental health and coping [65]. A recent review by Bakker et al [65] demonstrates that mental health smartphone apps can be effective for managing psychological problems, including depression and anxiety [66]. Fewer studies describe the integration and impact of psychological theories and psychotherapeutic techniques into the design and development of mobile apps for adolescent weight management [67].

Finally, the app interface should be simple and interactive, enjoyable to use, and intuitive. HCPs stressed the importance of designing the app to be very simple to use, whereas adolescents highlighted the importance of designing the user experience to be interesting, fun, and entertaining. Turner et al [25] reviewed usability studies of mobile technologies for pediatric obesity, which have provided some direction to future designs of app interface and the user experience. Ease of use and reduced demands on the user [68,69], reward-type incentives, social connection, and multiplayer gaming were proposed as major usability requirements by participants and researchers [20]. Studies have also pointed to motivational and personalized feedback as important features of the user’s interaction with the device [70]. Hamine et al [21] describe automation, motivational content, health challenges, and data analytics as main features of most mhealth tools for chronic disease management. In summary, the features discussed should be effectively integrated within the app’s program architecture to ensure that usability is simple, convenient, interesting, and entertaining. Potential app designs can employ strategies for gamification and social engagement to drive behavior change, while customizing app functionality to the user’s goals and preferences.

### Limitations

This study presents preliminary findings toward the development of a mobile app for weight and health management using a qualitative research design. Hence, findings reflect the subjective perspectives of adolescents with complex health care needs in specialized clinical programs and should not be generalized to adolescents in the general population who are not as intensively engaged with the health care system and may not benefit from enhanced health care support through a mobile app. More research using quantitative and qualitative research designs should be used to substantiate these results and to evaluate and improve on proposed app designs. Second, it should be noted that the majority of adolescent participants in this study were girls, potentially limiting proper description of the unique needs of boys in a mobile app for weight and health management. Further work is needed to characterize the views of adolescent boys and actively involve boys and girls in the design and implementation of the tool.

### Conclusions

The findings presented here describe the core functional components of a mobile app for weight and health management in adolescents with complex health needs. These findings highlight the lack of such a tool for adolescents in frequent contact with specialized health care services who could potentially benefit the most from more accessible health support through this technology. Remaining questions to be addressed center on how to best design these features into an app that is useful, interesting, as well as engaging for adolescents. Future directions for this research will be to develop the app and conduct iterative cycles of usability and feasibility testing before undergoing a pilot RCT to examine preliminary measures of effectiveness.

## Abbreviations

BMI: body mass index
HCP: health care provider
mHealth: mobile health
PA: physical activity
RCT: randomized controlled trial
SMS: short message service
UI: user interface
